# Curing Sickle Cell Disease by Allogeneic Hematopoietic Stem Cell (HSC) Transplantation Toward In Vivo HSC Gene Therapy

**DOI:** 10.3390/genes16111367

**Published:** 2025-11-11

**Authors:** Rina Kansal

**Affiliations:** 1 Molecular Oncology and Genetics, Diagnostic Laboratories, Versiti Blood Center of Wisconsin, Milwaukee, WI 53233, USA; rinakansal@msn.com; 2 Department of Pathology and Anatomical Sciences, The University at Buffalo, Buffalo, NY 14260, USA

**Keywords:** sickle cell disease, genetic therapy, CRISPR, hematologic diseases, pediatrics, therapeutics, hemoglobin, hematopoietic stem cell transplant, myeloablative conditioning, secondary neoplasms

## Abstract

Sickle cell disease comprises a group of prevalent inherited disorders defined by an underlying sickle cell allele that forms sickle hemoglobin. The incidence of this disease is rising, with more than 500,000 children born with it globally. The disease carries significant morbidity and mortality. Its only curative treatment was an allogeneic hematopoietic stem cell (HSC) transplant (HSCT) until late 2023, when two one-time gene therapies were approved for treating patients aged 12 years or older with severe sickle cell disease. This work aims to inform readers about these two gene therapies: one lentiviral-based and the other nonviral. The latter is based on the Nobel Prize-winning discovery of clustered, regularly interspaced, short, palindromic repeats (CRISPR)/CRISPR-associated (Cas)9 proteins and single-guide RNA (sgRNA)-based genome editing. Both approved gene therapies require an autologous HSCT with ex vivo genetically edited autologous hematopoietic stem and progenitor cells. Therefore, access to these gene therapies is limited to specialized centers with expertise in HSCTs. This review is meant for students, researchers, and clinical practitioners. It explains the basis for both approved gene therapies, their mechanisms of action, differences, risks, and other lentiviral-based and CRISPR-Cas9-based ex vivo gene therapies for sickle cell disease in clinical development. Additionally, it discusses the current state of preclinical studies for in vivo HSC gene therapy for sickle cell disease, which utilize advanced genome editing technologies developed after CRISPR-Cas9-sgRNA-based genome editing. In vivo HSC gene therapy, after it is clinically developed, would eliminate the need for an HSCT in receiving gene therapy and vastly increase access for numerous patients worldwide, even in low-income countries with the most significant disease burden.

## 1. Introduction

Sickle cell disease comprises a group of inherited hemoglobin disorders prevalent worldwide, including in sub-Saharan Africa, the Mediterranean, Western Europe, the Middle East, Asia, North America, and Latin America [1,2,3]. The disease has high morbidity due to the pathophysiological mechanisms caused by hemoglobin S (HbS), which include sickle-shaped red blood cells, painful and recurrent vaso-occlusive crises, and multi-organ damage, including affecting the brain, kidneys, and heart due to ischemia and reinfusion injury, in addition to hemolytic anemia [1]. The molecular abnormality underlying the production of hemoglobin S is a base pair substitution in codon 6 of the β-globin gene, from adenine (in GAG codon) to thymine (in GTG codon). This single point mutation in codon 6 changes the amino acid from glutamic acid to valine, leading to HbS, which polymerizes when deoxygenated to cause the pathologic effects of sickle cell disease [1]. Thalassemia syndromes are a group of inherited hemoglobinopathies that are also prevalent worldwide and are characterized by deficient or absent synthesis of one or more globin chains [4,5]. β-Thalassemia syndromes are caused by genetic mutations that lead to reduced (β^+^-thalassemia) or absent (β^0^-thalassemia) synthesis of β-globin chains [5,6]. β-Thalassemia syndromes show variable clinical severity, with their pathophysiology attributed to anemia, ineffective erythropoiesis, and hemolytic anemia, including transfusion-independent and transfusion-dependent forms [5,7,8].

Individuals with sickle cell disease may have different genotypes; each includes at least one sickle cell mutation (β^S^) in the β-globin gene, combined with a second mutation in the same or in a different locus of the β-globin gene. If both mutations in the β-globin gene correspond to the β^S^ allele (homozygosity for the β^S^ allele), it leads to sickle cell anemia, the most common disease form [1]. Compound heterozygotes of sickle cell disease harbor a sickle cell (β^S^) allele and a variant β-globin allele that results in hemoglobin C, D, E, or β-thalassemia at the second β-globin locus [1]. Individuals with sickle cell trait have one sickle cell allele and one normal allele [2,9]; they are often asymptomatic but may have adverse complications related to exercise, high altitude, chronic kidney disease, venous thromboembolism, and a risk of developing renal medullary carcinoma [10].

In 2008, hemoglobin disorders were found to be a significant health problem in 71% of 229 countries: those 71% countries accounted for 89% of all worldwide births. Among all affected infants, 83% were due to sickle cell disorders and 17% due to thalassemia syndromes [11]. From 2000 to 2021, the number of people living globally with sickle cell disease increased 41·4% (38·3–44·9), from 5·46 million (4·62–6·45) in 2000 to 7·74 million (6·51–9·2) in 2021 [12]. During the same time, the total number of births increased by 13.7% globally to an estimated 515,000 (425,000–614,000) births annually [12]. A systematic review of publications from 2010 to 2022 showed the highest prevalence of sickle cell disease per 100,000 individuals in Africa (*n* = 788), followed by the Middle East (*n* = 212), India (*n* = 128), and Europe (*n* = 28) [3]. Sickle cell trait prevalence per 100,000 individuals showed a similar pattern, with the highest in Africa (*n* = 17,690), followed by the Middle East (*n* = 2429), India (*n* = 2193), and Europe (*n* = 212) [3]. The trait protects against severe malaria and is highly prevalent in countries with endemic malaria [1,2,12]. The number of individuals with sickle cell disease in the USA is unknown. Still, at least 100,000 persons in the USA are estimated to be affected, including 90% non-Hispanic Blacks and African Americans and 3–9% of Hispanic or Latino origin [13]. Sickle cell trait is reported in 2.5 million U.S. individuals [14].

Sickle cell disease also carries a high global mortality burden. The mortality rate for children under five years old is as high as 90% in some cross-sectional studies in Africa [15] (reviewed in [12]). Severe sickle cell disease causes functional asplenia from a very young age, leading to susceptibility to infections, especially with encapsulated bacteria [16], a major cause of mortality in early childhood in low-income countries [17,18]. The Global Burden of Diseases, Injuries, and Risk Factors Study distinguished cause-specific mortality from total mortality of sickle cell disease among patients with hemoglobinopathies from 2000 to 2021 across 204 countries [12]. Cause-specific mortality was recorded only when sickle cell disease was listed as the single underlying cause based on the International Classification of Diseases codes. In contrast, total sickle cell disease mortality included all deaths in patients with sickle cell disease, regardless of the recorded cause. Globally, in 2021, total sickle cell disease mortality was found to be 11 times higher, at 376,000 (303,000–467,000), than cause-specific mortality, which was 34,400 (25,000–45,200) for all ages [12]. In children under five years old, the total sickle cell disease mortality of 81,100 (58,800–108,000) deaths ranked 12th, in contrast with cause-specific mortality, which ranked 40th among causes of death [12].

Sickle cell disease lowers life expectancy by about thirty years worldwide, with a greater impact in lower-income countries, where infant mortality is a major concern [19,20]. During 2017–2018, the projected life expectancy and quality-adjusted life expectancy for sickle cell disease in the USA were 54 and 33 years, respectively, compared to 76 and 67 years, respectively, in those without the disease [21]. Among 94,616 U.S. individuals with sickle cell disease not treated by a transplant, life expectancy at birth was 52.6 years (95% confidence interval (CI): 51.9–53.4) [22]. Even in U.S. academic centers, the median survival of individuals receiving comprehensive care for sickle cell disease is ≥20 years less than that of U.S. African Americans [23]. Age-adjusted mortality rates per 1,000,000 U.S. individuals are highest in African Americans (11.91; 95% CI: 11.69–12.14), followed by non-Hispanic (1.93; 95% CI: 1.85–2.00), Hispanic (0.38; 95% CI: 0.31–0.45), and White patients (0.09; 95% CI: 0.08–0.10) [24]. High social vulnerability index scores, which consider socioeconomic status, household composition, disability, minority status, language, housing type, and transportation, are associated with high mortality rates in adult U.S. patients with sickle cell disease [24].

Early diagnosis, ideally through newborn screening (NBS), access to treatments such as hydroxyurea and blood transfusions, antibiotic prophylaxis, and pneumococcal vaccinations are essential to improve outcomes for individuals with this disease, along with point-of-care diagnostic tests, genetic screening, and preventive measures like transcranial Doppler screening [25]. The World Health Organization estimates that roughly 5% of the global population carries a variant allele for hemoglobin disorders, primarily sickle cell disease and thalassemia, with over 300,000 new births annually affected by severe hemoglobin disorders worldwide [26]. When both parents carry a sickle cell allele, there is a 25% chance their child will have sickle cell disease. Testing before marriage or pregnancy to identify at-risk couples, followed by genetic counseling to inform them about the risks, treatments, and options, can help prevent an increase in the disease burden [26].

NBS allows for diagnosing sickle cell disease before symptoms appear, enabling necessary care such as vaccinations and infection prevention, which reduces morbidity and mortality. Universal NBS for sickle cell disease has been implemented in many high-resource countries, including the USA and several European nations [27,28], with ongoing efforts toward its implementation in low-income countries with a high disease burden [29,30,31,32]. In the USA, universal NBS for sickle cell disease was recommended in 1987 and adopted by all states in 2006 [33]. However, states differ in the platforms used for initial screening, confirmatory testing, reporting, and communicating results to families [27], with inconsistencies and barriers identified in these practices and in referring affected newborns and their families for sickle cell disease care [34,35]. The positive impact of NBS in reducing morbidity and improving the quality of life in screened infants, compared with diagnosing sickle cell disease after symptoms occur, has been shown in studies from Spain [36], Senegal [37], and Canada [38], indicating that universal NBS should be prioritized globally.

Until 2017, the only treatment options for sickle cell disease were transfusions and hydroxyurea, even in high-income countries. These were later followed by drug approvals for L-glutamine, crizanlizumab, and voxelotor [39]. Hydroxyurea increases fetal hemoglobin levels, which prevents the polymerization of HbS, and increases hemoglobin and mean red cell volumes [40,41]. Voxelotor binds to the *N*-terminal valine of the α chain of hemoglobin to increase its oxygen affinity, thus inhibiting HbS polymerization [42,43]. Voxelotor was globally discontinued by the manufacturer in September 2024, as risks outweighed benefits due to the occurrence of deaths in several treated patients [44]. Hydroxyurea has a long-standing record of safety and efficacy, having been FDA-approved for treatment in 1998, and is considered a gold standard in the management of sickle cell disease [41]. The availability of hydroxyurea is variable in countries other than high-income countries: it is readily available and even dispensed at no cost to patients with sickle cell disease in India [45]. However, in African nations, limited availability, access, frequent supply disruptions, education and awareness, and cost considerations hinder treatment [46,47]. A clinical trial showed hydroxyurea treatment to be feasible, effective, and safe, reducing the rates of painful events, infection, malaria, transfusion, and death in children with sickle cell disease in sub-Saharan Africa, highlighting the need for wider drug availability in conjunction with NBS as important goals to improve disease outcomes [48].

The only current way to cure sickle cell disease is by replacing the diseased hematopoietic stem and progenitor cells (HSPCs) with healthy hematopoietic stem cells. Those healthy cells could be obtained from a donor other than the patient, as in an allogeneic hematopoietic stem cell transplant (HSCT). Alternatively, gene therapy approaches based on viral integration of genetically modified cells have been studied for over three decades and have been developed to treat several genetic diseases, including sickle cell disease and β-thalassemia (reviewed in [49,50]). Gene therapy uses genetic materials, most commonly DNA or RNA, to prevent or treat disease [51]. The following three approaches are commonly used for gene therapies: (a) gene addition: a functional gene with the instructions to make the needed protein is added to the host cells, (b) gene silencing: the inserted genetic material inactivates or prevents the activity of a gene present in the cell, and (c) gene editing: genetic material is delivered to edit or change the DNA sequence within the affected gene inside the cell [51]. The genetic materials are delivered to the host cells by viral vectors, which are modified viruses with the viral genes removed or replaced by the therapeutic genetic material packaged in the virus shell [51]. The viral vector is delivered either in vivo, i.e., directly into the patient’s body, or ex vivo, i.e., into cells removed from the body to create genetically modified cells, which are subsequently returned to the patient [51].

Hematologic genetic diseases that may be treated by gene addition or gene editing include (a) those involving deficiencies of plasma proteins, such as in hemophilia, and of other enzymes, such as lysosomal storage disease, and (b) those affecting blood cells, such as erythrocytes in hemoglobinopathies, leukocytes in immune disorders, and hematopoietic stem cells in Fanconi’s anemia [52]. Adeno-associated viruses persist but minimally integrate and have been used to deliver therapeutic genetic materials in vivo to tissues such as hepatocytes, neurons, muscle cells, and retinal cells [52]. In contrast, ex vivo gene therapies have used retroviral and lentiviral vectors that integrate into the cell genome to persist long-term after the genetically modified cells are transplanted back into the patient [52]. In the last two decades, zinc finger nucleases (ZFNs) were the first gene editing technologies to move into clinical trials, followed by transcription activator-like effector (TALE) nucleases (TALENs), as previously reviewed [53,54,55,56]. ZFNs and TALENs are programmable complex engineered proteins comprising the *Flavobacterium okeanokoites* enzyme number one (FokI ) nuclease to cut the DNA [57]. However, a new zinc finger or TALE module rearrangement in ZFNs or TALENs must be manufactured for every new target sequence [57].

The discovery of clustered, regularly interspaced, short, palindromic repeats (CRISPR)/CRISPR-associated (Cas)9-based genome editing guided by a single-guide RNA (sgRNA) in 2012 [58], as reviewed in 2014 [59], led to the rapid development of precise CRISPR-Cas9-based gene editing tools for applications in medicine, science, and agriculture [60]. Less than a decade after its discovery in 2012, CRISPR-Cas-sgRNA-based gene editing entered human clinical trials for treating patients with sickle cell disease [61], with results published first in 2021 [62]. The first lentiviral-based gene therapy approved in the USA was for transfusion-dependent β-thalassemia [63,64]. Subsequently, in 2023, the first CRISPR-Cas9-sgRNA-based gene therapy was approved as a one-time curative treatment for patients with sickle cell disease and β-thalassemia in the UK in November [65], followed by approvals by the United States Food and Drug Administration (FDA) [66] and in Europe in December, as previously reviewed [56]. This approval is a significant landmark in medicine, hopefully leading to further advances that benefit patients. This review is meant for students, researchers, and clinical practitioners, focused on explaining the basis of lentiviral-based and non-viral CRISPR-Cas9-based gene therapies for the curative treatment of sickle cell disease. Future possibilities and challenges to enhance the delivery of curative gene therapies and increase access to affected patients with sickle cell disease worldwide are briefly discussed.

## 2. Curing Sickle Cell Disease: Allogeneic Hematopoietic Stem Cell Transplantation or Gene Therapies, Lentiviral-Based or Nonviral CRISPR-Cas9-Based

### 2.1. Hematopoietic Stem Cell Transplantation

An HSCT is a standard treatment for many neoplastic and non-neoplastic diseases [67,68,69]. Its procedure requires multiple steps, including (a) priming or mobilization to release stem cells from the bone marrow into the peripheral blood, (b) stem cell harvest or collection of stem cells from the patient’s (autologous HSCT) or donor’s (allogeneic HSCT) bone marrow by aspiration or peripheral blood by apheresis, (c) conditioning treatment, a preparatory regimen, including chemotherapy, radiotherapy, or both to destroy the patient’s original bone marrow and prepare it for infusion of new stem cells, and (d) stem cell infusion, which are followed by engraftment [68].

### 2.2. Allogeneic Hematopoietic Stem Cell Transplantation in Sickle Cell Disease

With the current treatments for sickle cell disease, including vaccination, antibiotic treatment, parent education, and hydroxyurea starting in infancy, the mortality for children has decreased. But mortality for adults has only shifted to an older age since the conventional treatments do not prevent progressive organ damage [69]. Therefore, an allogeneic HSCT using a human leukocyte antigen (HLA)-matched sibling donor is considered standard of care; it is a potentially curative treatment that should be offered before complications occur in sickle cell disease [69,70]. In 1000 patients with sickle cell disease who received an allograft from 1986 to 2013, the median age at transplantation was 9 years; 87% and 13% received a myeloablative or reduced-intensity conditioning regimen, respectively, and 5-year event-free survival and overall survival were 91.4% and 92.9%, respectively [71]. Myeloablative conditioning in adults with sickle cell disease can be toxic; nonmyeloablative allogeneic HSCT has been successful for adult patients with a matched related donor with a high cure rate and improved quality of life [72,73,74,75].

Still, an allogeneic HSCT itself has the potential for serious complications, including graft failure, graft versus host disease (GVHD), and death. In a study of 736 patients with sickle cell disease who underwent HSCT from an HLA-matched sibling donor between 1986 and 2017, overall survival and event-free survival were better in younger patients [76]. Further, in patients with ages >15 years, acute and chronic GVHD incidence increased to 17% and 20%, respectively, emphasizing the importance of early referral to HSCT [76]. In 996 patients in the USA with sickle cell disease who received an allograft between 2008 and 2017, event-free survival was improved in patients aged ≤12 years and those with an HLA-matched sibling donor [77]. The clinical outcomes were worse in HSCT from haploidentical related donors, matched unrelated donors, and mismatched unrelated donors [77].

However, an HLA-matched donor is only available in a small proportion (estimated 18%) of patients with sickle cell disease [78], limiting access to HSCT [77,78]. The frequency of donors by ethnic origin in the National Marrow Donor Program (NMDP) Registry is reported as follows: 11% Blacks, 13% Hispanic or Latino, 1% Native Americans, 12% Asian, 57% White, 9% multiple races, and 3% unknown [79]. Since 90% of U.S. patients with sickle cell disease are non-Hispanic Blacks and African Americans, the underrepresentation of donors of African American ancestry in the donor registry limits the ability to find a donor. In 2024, 14,000 African Americans aged 18–24 joined the NMDP Registry. Still, many more African American donors are needed, especially for sickle cell disease [80]. Even in the USA, less than 1% of the population with sickle cell disease has received a transplant [81]. Most transplants in the USA and Europe have used HLA-matched sibling donors. The patient registries of the Center for International Blood and Marrow Transplant Research (CIBMTR) (*n* = 627) and the European Blood and Marrow Transplant (EBMT) (*n* = 611) have shown 3-year overall survival rates of 91% and 95%, respectively [82]. More than 90% of patients with sickle cell disease receiving HLA-identical sibling donor grafts are cured, with limited complications [82]. A recent multicenter study of 56 patients with sickle cell disease who underwent HLA-haploidentical bone marrow transplantation at a median age at enrollment of 22.8 years (range, 15.5 to 43.2 years) reported adverse events, including a 2-year chronic GVHD rate of 22.4%, along with 2-year event-free and overall survival rates of 88.0% and 95.0%, respectively [83].

#### 2.2.1. Risk of Developing a Secondary Malignancy After an Allogeneic Hematopoietic Stem Cell Transplant

Further, there is also the risk of developing a malignancy after an allogeneic HSCT for sickle cell disease. A 10-year incidence of leukemia or myelodysplastic syndromes in 1.7% and any secondary neoplasm in 2.4% were observed in 1096 transplants for sickle cell disease between 1991 and 2016 [84]. In 22 patients with a secondary neoplasm, the median age at transplantation was 19 years compared with 11 years for 1074 patients with no neoplasm [84]. In transplanted patients who developed a secondary neoplasm, the predominant conditioning regimen was non-myeloablative, low-dose total body irradiation [84], with the conditioning regimens defined according to published criteria [84,85]. The predominant donor type in the patients with a secondary neoplasm was a haploidentical relative, with peripheral blood as the predominant graft [84].

Conversely, the following characteristics were predominant in transplanted patients who did not develop a secondary neoplasm: a myeloablative conditioning regimen of busulfan with cyclophosphamide or fludarabine, an HLA-matched sibling donor, and bone marrow as the graft [84]. The authors hypothesized that the persistence of host cells triggered by low-dose radiation may have caused the myeloid malignancies; however, pre-existing clonal mutations could not be studied in that cohort [84].

In another cohort of 120 patients who underwent HSCT, eight patients aged 19–53 (median 37) years at transplant developed a hematologic malignancy between 4 months and 9 years post-transplant; five (62.5%) of these eight had received a matched sibling donor transplant, and three (37.5%) had received a haploidentical transplant [86]. Five patients developed a myelodysplastic syndrome/leukemia and died; all five had persistent autologous hematopoiesis with graft failure (*n* = 4) and low-donor chimerism with imminent graft failure (*n* = 1) [84,86].

### 2.3. Autologous Hematopoietic Stem Cell Transplantation Using Genetically Modified Autologous Hematopoietic Stem and Progenitor Cells, i.e., Gene Therapy in Sickle Cell Disease

In contrast to allogeneic HSCT, autologous hematopoietic stem cell transplantation using genetically modified autologous HSPCs, i.e., gene therapy, eliminates the possibilities of serious immune complications and the need to find an HLA-matched sibling donor [49]. Genetic modification of HSPCs for autologous transplantation was developed earlier over three decades by viral integration methods and has been developed much more rapidly in the past decade by nonviral CRISPR-Cas9-based gene editing [49]. Hemoglobinopathies due to β-globin disorders such as β-thalassemia and sickle cell disease are prevalent worldwide. Gene therapy has the potential for great clinical benefit to these patients, which led to early efforts to develop these curative therapies using lentiviral vectors.

#### 2.3.1. Lentiviral-Based Gene Therapy Approved for Treating Sickle Cell Disease

The first gene therapy success in hemoglobinopathies was witnessed in 2010 when an 18-year-old male with severe transfusion-dependent β-thalassemia and no available HLA-matched donor was shown to become transfusion-independent for 21 months at 33 months after receiving the gene therapy [87]. This gene therapy was developed using a self-inactivating lentiviral vector encoding a T87Q variant of adult β-globin with anti-sickling properties, representing an example of gene addition [87]. In 2022, the FDA approved the lentiviral gene product betibeglogene autotemcel (Zynteglo^TM^) for treating pediatric and adult patients with β-thalassemia based on the results showing transfusion independence in most patients enrolled in the clinical trials [64].

For sickle cell disease, gene therapy was successful in 2017 in a 13-year-old boy with the disease who was treated with a similar lentiviral vector-based drug product LentiGlobin BB305 encoding the human β-globin gene T87Q (β^A-T87Q^) to produce the anti-sickling hemoglobin variant HbA^T87Q^ [88]. This drug product was subsequently used in a single-arm, 24-month multicenter clinical trial in the USA in 45 patients aged 12–50 years with sickle cell disease and a history of at least four vaso-occlusive events in the 24 months before enrollment (ClinicalTrials.gov number, NCT02140554). The results showed a complete resolution of vaso-occlusive events and sustained hemoglobin variant T87Q production [89].

#### 2.3.2. Causation of Malignancy After Treatment of Sickle Cell Disease with the Lentiviral-Based Gene Therapy

Of note, a hematologic malignancy, acute myeloid leukemia, developed in two patients in the LentiGlobin BB305 trial after three years and five and a half years of receiving the lentiviral gene therapy [90,91,92]. Both patients who developed myeloid malignancy were enrolled in the initial group in the clinical trial, Group A, which comprised seven patients [89]. This initial group had received a lentiviral-induced gene therapy product manufactured by an earlier protocol that had led to fewer CD34-positive long-term HSPCs, which led to increased proliferative stress [89]. There was suboptimal expression of the antisickling HbA^T87Q^ and an inadequate therapeutic response, leading to further hematopoietic stress after the therapy was given, with the possibility for mutations to develop [89,90]. In both patients, cytogenetic and molecular abnormalities were found in the neoplastic cells at the diagnosis of malignancy. The first reported patient harbored monosomy 7 and structurally abnormal chromosome 19p in 8 of 20 metaphases, along with mutations in *RUNX1*, *KRAS*, and *PTPN11* [90]; in the second patient, monosomy 7 was observed in 70% of cells and partial loss of 11p involving *WT1* in 50% of cells, along with mutations in *RUNX1* and *PTPN1* [91]. No genetic abnormalities were identified in the pre-gene therapy archived bone marrow samples in either patient [90,91,92]. The viral vector integration site was absent in the blast cells in the first reported patient [90]. Although the vector was identified in the blast cells in the second patient, there was evidence to show that the leukemia occurred independently of the lentiviral insertion site at a noncoding region of *VAMP4*, a gene with no known role in cellular proliferation or oncogenesis [91].

Moreover, patients with sickle cell disease have a two to ten times higher risk of developing a malignancy compared to the general population [93,94]. That inherent risk, combined with the increased risk of developing a malignancy following myeloablative conditioning by busulfan, and the increased post-transplant hematopoietic stress due to lower numbers of CD34-positive HSPCs and suboptimal therapeutic response, were considered as the likely reasons for the development of malignancy in both patients; mutagenesis due to viral insertion was considered unlikely [90,91,92]. The FDA approved this gene therapy in December 2023, with a black box warning for this lentiviral gene therapy for the possibility of developing blood cancers [66]. Patients who received the LentiGlobin BB305 gene therapy are to be followed up for 15 years post-therapy to evaluate the therapy’s long-term safety and efficacy (https://clinicaltrials.gov/study/NCT04628585, accessed on 1 September 2025).

It is worth noting that an extensive study of 74,190 whole-genome sequences did not show a detectable variation in the rate of clonal hematopoiesis or clone properties in sickle cell disease compared to controls [95]. However, an exome sequencing study, a method with a greater depth of sequencing than whole genome sequencing, showed increased occurrence of clonal hematopoiesis in patients with sickle cell disease than in individuals without sickle cell disease [96]. As previously noted [94,95], the increased risk of developing leukemia in patients with sickle cell disease who are untreated with cellular therapies is likely related to the stress caused by hemolysis and chronic inflammation in the bone marrow. Still, further studies are required to evaluate the risks of developing a hematologic malignancy after receiving gene therapy.

### 2.4. Comparing Allogeneic Hematopoietic Stem Cell Transplantation with Autologous Hematopoietic Stem Cell Ex Vivo Gene Therapy in Sickle Cell Disease

Allogeneic HSCT and autologous HSCT with genetically edited HSCs are different curative treatments for sickle cell disease. However, they have not yet been compared in a clinical trial. Therefore, a systematic review compared the two treatments, including allogeneic HSCT from all HSC sources, lentiviral gene therapy, gene editing with targeted endonucleases, and all conditioning regimens [97]. All Phase I–III clinical trials, case reports, or series involving >one patient at any age who underwent HSCT or gene therapy to cure sickle cell disease were included in the review. Literature searching identified 948 titles until 1 June 2020, and an additional 15 titles by searching reference lists and peer review. A total of 56 studies were included, representing 1198 patients who underwent HSCT (*n* = 1158) or gene therapy (*n* = 40; all lentiviral). The donor HSC source was reported for 1032 HSCT patients: HLA-matched related donor (*n*  =  821), matched unrelated donor (*n*  =  73), haploidentical (*n*  =  127), and HLA-mismatched unrelated donor (*n*  =  11) [97]. The authors noted the presence of secondary malignancies in both treatment groups, including five patients following allogeneic HSCT for sickle cell disease over more than 3800 years of patient follow-up. All five patients developed hematolymphoid neoplasms, including three myelodysplastic syndromes/acute myeloid leukemia and two post-transplant lymphoproliferative disorders [97]. The conditioning regimens were reported in only two of the five patients who had developed high-risk MDS at 2 and 5 years post-transplant. Both had received haploidentical donor HSCT with non-myeloablative conditioning, including total body irradiation and alemtuzumab. One of these two patients had also received post-transplant cyclophosphamide [97]. Since two patients after lentiviral gene therapy also developed a hematologic malignancy in similar time frames of 3 and 5 and a half years post-transplant, as described in Section 2.3.2, the authors raised concerns about conditioning regimens and highlighted the need for long-term follow-up as recommended by the FDA [97,98].

That systematic study did not include the 22 patients with secondary neoplasms (*n* = 15, myelodysplastic syndrome/leukemia, and *n* = 7, solid tumors) identified in a U.S. observational cohort of 6631 person-years (*n* = 1096 patients) mentioned in Section 2.2.1 [84]. The median age at diagnosis of a neoplasm was 26 (range 4–57) years, and the median time to develop neoplasms was 40 (range 9–196) months in that study [84]. The risks for developing a myelodysplastic syndrome/leukemia or any other secondary neoplasm were higher with low-intensity (nonmyeloablative) conditioning regimens, which result in mixed-donor chimerism. Therefore, selecting conditioning regimens that result in full-donor chimerism may partly reduce the higher risk of developing a secondary neoplasm [84].

Table 1 compares allogeneic hematopoietic stem cell transplantation with gene therapy, i.e., autologous hematopoietic stem cell transplantation with genomically edited HSPCs, in treating sickle cell disease.

**Table 1:** Comparison of allogeneic hematopoietic stem cell transplantation with gene therapy in treating sickle cell disease.

	Allogeneic Hematopoietic Stem Cell Transplantation	Autologous Hematopoietic Stem Cell Ex Vivo Gene Therapy
Source of transplanted HSPCs	Donor, preferred HLA-matched	Self (autologous HSPCs); no donor
Availability of donor	Only up to 30% of patients requiring a transplant have an HLA-matched donor	Not applicable
Genome editing of HSPCs performed	None	Yes, via Lentiviral vector or Nonviral CRISPR-Cas9-single guide RNA
Risks of treatment		
Immunological complications	Yes	None
- Graft-versus-host disease, acute	Yes	No risk
- Graft-versus-host disease, chronic	Yes	No risk
Due to myeloablative conditioning (chemotherapy)	Yes, including treatment-related mortality, infertility, and increased risk of cancer	Yes, including treatment-related mortality, infertility, and increased risk of cancer
Graft failure	Yes, possible	Not applicable
Donor-derived leukemia	Yes	No risk
Secondary malignancy reported	Yes; 3 cases per 1000 person-years [84]	Yes, reported in lentiviral-based therapy, not in CRISPR-Cas9-based therapy
Off-target toxicity risk present	Not applicable	Yes
Costs of therapy	Variable, up to about $0.5 million	$2.2 to $3.1 million
Efficacy of therapy	Event-free survival depends on patient age (better in <12 years), donor type, and conditioning [77]; overall 3-year survival 91% and 95% in CIBMTR and EBMT registries [82]	High chance (>95%) of eliminating vaso-occlusive crises and disease symptoms
Accessibility	Also available for ages under 12 years	Only available for ages 12 years and older with recurrent vaso-occlusive events
	Only available in specialized centers with access to expertise in HSC transplantation	Only available in specialized centers with access to expertise in HSC transplantation
Follow-up	Established treatment for decades	Extensive follow-up required for long-term evaluation of efficacy and safety

HSPCs, hematopoietic stem and progenitor cells; HLA, human leukocyte antigen; CIBMTR, Center for International Blood and Marrow Transplant Research; EBMT, European Blood and Marrow Transplant; HSC, hematopoietic stem cell.

## 3. Ex Vivo CRISPR-Cas9-Based Gene Therapy Approved for the Treatment of Severe Sickle Cell Disease

The CRISPR-Cas9 system is an immune defense mechanism in microbes that protects against viruses and bacteriophages. A chronological history of its discovery was recently reviewed, from a sequence of unknown significance in 1987 to the understanding that it is a natural microbial defense mechanism and the discovery of targeted CRISPR-Cas9 single-guide RNA-based genome editing in humans [56].

The CRISPR-Cas9-based gene editing clinical trial that led to the FDA approval in 2023 treated 44 patients aged 12 to 35 years with sickle cell disease having a history of at least two severe vaso-occlusive crises in each of the two years before screening (ClinicalTrials.gov number, NCT03745287). Results from this clinical trial showed that there were no vaso-occlusive crises for at least 12 months in 29 (97%) of 30 patients with sufficient follow-up, and all 30 (100%) of the patients were free of hospitalizations for a vaso-occlusive crisis for at least twelve consecutive months [99]. No cancers developed in any patient, and the therapy’s safety profile was similar to that of myeloablative busulfan conditioning and autologous hematopoietic stem cell transplantation [99]. The post-therapy amelioration of disease symptoms was described as a “miracle” by the first patient with sickle cell disease treated with this gene therapy [100]. Patients who received the CRISPR-Cas-based gene therapy are to be followed up long-term to determine the long-term safety and efficacy of the therapy (https://clinicaltrials.gov/study/NCT04208529, accessed on 1 September 2025).

### 3.1. The Normal Hemoglobin Switch at Birth and the Protective Effect of Fetal Hemoglobin in Sickle Cell Disease

Hematopoietic stem cells proliferate and differentiate to produce the hemoglobin-carrying mature red blood cells. At birth, fetal hemoglobin (HbF) transitions to adult hemoglobin, requiring a switch from producing γ-globin in the fetus to β-globin after birth. This switch is controlled by the expression of the B-cell lymphoma/leukemia 11A (*BCL11A*) gene, which normally represses fetal hemoglobin at birth. The first step in this discovery was the genome-wide association studies in 2007 that linked *BCL11A*, located on chromosome 2, to regulating fetal hemoglobin production [101]. *BCL11A* is also important for normal immune and neurodevelopmental functions, with pathogenic loss-of-function mutations in *BCL11A* known to be harbored in neurodevelopmental disorders [102,103]. *BCL11A* was shown to be developmentally regulated and expressed only in adult-stage erythroblasts, i.e., precursors of red blood cells, wherein *BCL11A* repressed the erythroid promoter of fetal hemoglobin and increased the levels of fetal hemoglobin without affecting the cellular differentiation state [104]. Inactivating *BCL11A* in genetically engineered mice reversed the pathologic effects of sickle cell disease in mice, providing further proof of *BCL11A* being a therapeutic target [105]. Further studies showed that *BCL11A* directly binds to embryonic and fetal-expressed globin promoters via a preferred TGACCA DNA motif, which is duplicated in γ-globin promoters, to repress the promoter and control the fetal-to-adult hemoglobin switch, thus providing a direct therapeutic target for β-thalassemia and sickle cell disease [106]. Mutations in the distal DNA motif, which prevent binding by *BCL11A*, occur rarely as the cause of hereditary persistence of fetal hemoglobin [106].

The human erythroid lineage-specific enhancer of *BCL11A*, located in intron 2 of *BCL11A*, contains three DNase I hypersensitive sites, called h + 55, h + 58, and h + 62, based on distance in kilobases from the transcriptional start site [107]. Disrupting critical sequences in the erythroid-specific enhancer of the *BCL11A* gene in a cell line-derived HSPCs by using CRISPR-Cas9-sgRNA-based genome editing led to a selective reduction in *BCL11A* expression in erythroid cells and reactivation of γ-globin with increased HbF in red blood cells [108]. These studies validated the erythroid enhancer of *BCL11A* as a target for fetal hemoglobin reinduction [108]. In preclinical studies, deleting the erythroid enhancer of *bcl11a* in mice did not affect the gene’s expression in non-erythroid lineages [109]. Another study used ZFNs in human bone marrow-derived CD34+ HSPCs for targeted disruption of the *BCL11A* erythroid enhancer at exon 2 or at the GATAA motif; disrupting both targets upregulated fetal globin expression to levels that would inhibit HbS polymerization. However, complete inactivation of *BCL11A* by disrupting exon 2 also adversely affected erythroid enucleation, which was not affected by the disruption of only the GATAA motif [110]. Subsequently, patient-derived hematopoietic stem cells modified by CRISPR-Cas-sgRNA-based cleavage within a GATA1 binding site at the +58 *BCL11A* erythroid enhancer were shown to achieve therapeutic levels of fetal hemoglobin and resist sickling in patients with sickle cell disease, and restoration of globin chain balance in patients with β-thalassemia [111].

The level of fetal hemoglobin and the presence of coexisting thalassemia are two factors that affect the severity of sickle cell disease [1]. Increased levels of HbF contribute to preventing the polymerization of HbS, thus minimizing its downstream pathologic effects [1,101]. The critical factor is the level of HbF in each erythrocyte, which influences the protective effect of HbF in sickle cell disease (reviewed in [112]). The mechanisms described above were harnessed to develop both the CRISPR-Cas9-based and lentiviral-based gene therapies for treating patients with sickle cell anemia.

### 3.2. The Mechanisms of Action of the FDA-Approved Lentiviral-Based Gene Therapy Lovotibeglogene Autotemcel (Lyfgenia ^TM^) and the CRISPR-Cas9-Based Non-Viral Gene Therapy Exagamglogene Autotemcel (Casgevy ^TM^) for Sickle Cell Anemia

**Figure 1:**
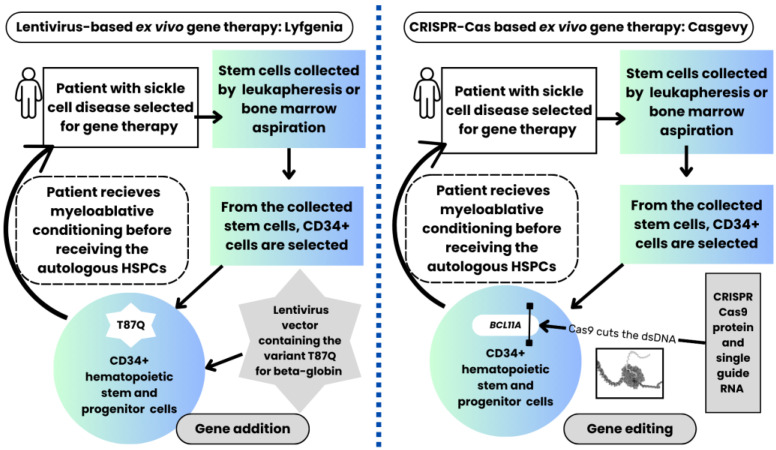
Gene therapy process in patients with sickle cell disease receiving the FDA-approved lentiviral vector-based gene therapy (**left** side of the image) compared with the CRISPR-Cas9-based gene therapy (**right** side). The patients undergo a similar process in both gene therapies. Both gene therapies require the collection of hematopoietic stem and progenitor cells (HSPCs) by leukapheresis or bone marrow aspiration, followed by selecting CD34+ stem cells in a Good Manufacturing Practice (GMP) facility. The CD34+ HSPCs are genetically modified ex vivo by either the lentivirus-based vector or non-viral CRISPR-Cas9-based gene therapy. However, the lentiviral-based gene therapy uses gene addition (the lentiviral vector contains the variant T87Q β-globin, which is added to the genome of the autologous HSPCs). In contrast, the CRISPR-Cas9-based therapy uses gene editing to modify the genome of the autologous HSPCs. As depicted, in the CRISPR-Cas9-based genome editing process, the Cas9 protein, which is precisely guided by a single RNA strand, cuts the double-stranded DNA (dsDNA) of the *BCL11A* gene in the autologous CD34+ HSPCs. The patient receives myeloablative conditioning before receiving the genome-modified autologous HSPCs (i.e., the gene therapy product), which engraft and repopulate the bone marrow with healthy cells. The different genome modification methods in both FDA-approved gene therapies lead to the production of fetal hemoglobin, which then occurs in the engrafted HSPCs-derived erythroid cells to ameliorate sickle cell disease.

As described above, reactivating the production of fetal hemoglobin effectively treats sickle cell disease. Both gene therapies approved in 2023 for sickle cell disease require autologous hematopoietic stem cell transplantation with autologous HSPCs that are genome-edited ex vivo and transfused to the same patient from whom the HSPCs were initially collected. The process for these two gene therapies is depicted in Figure 1.

The differences between these two therapies are related to how the production of fetal hemoglobin was reactivated for these two gene therapies, as described next.

### 3.3. Comparing the FDA-Approved Lentiviral-Based Gene Therapy (Lyfgenia ^TM^) with the CRISPR-Cas9-Based Non-Viral Gene Therapy (Casgevy ^TM^) for Sickle Cell Disease, Including Potential Risks of Both Gene Therapies

Lovotibeglogene autotemcel (Lyfgenia ^TM^) is a lentiviral-based gene therapy in which the genetic modification of HSPCs requires zinc finger nucleases. ZFNs are complex proteins genetically engineered by fusing two chimeric proteins, i.e., zinc finger DNA-binding proteins that target zinc finger domains present in the genome, which are fused with the cleavage domain of a unique Class II restriction enzyme FokI nuclease, derived from *Flavobacterium okeanokoites* [56,57]. The ZFNs are integrated into a self-inactivating lentivirus to produce the non-sickling T87Q variant of hemoglobin A. The virus is then transferred to the autologously collected CD34-positive HSPCs from the patient, which are then infused back into the patient after the patient undergoes myeloablative conditioning in preparation for the stem cell infusion. This gene therapy costs $3.1 million and carries a black box warning that this therapy carries a risk of developing blood cancer [66,113].

In contrast, exagamglogene autotemcel (Casgevy ^TM^) is a nonviral gene therapy in which gene editing is performed ex vivo using CRISPR-Cas9-sgRNA-based gene editing, based on the Nobel Prize-winning work by Doudna and Charpentier [58,59,114]. The single-guide RNA is a simple oligonucleotide strand that guides the Cas9 endonuclease to the precise site in the genome to be edited [58,59]. For treating sickle cell disease using Casgevy, the molecular scissor, Cas9, precisely cuts the double-stranded DNA for the GATA1 transcription factor binding domain in the erythroid enhancer of the *BCL11A* gene in the CD34+ HSPCs that were initially collected from the patient and enriched for CD34+ cells [99,115]. This gene therapy costs $2.2 million, less than the lentiviral-based gene therapy [113]. Table 2 compares the risks of the two gene therapies, as well as the costs, efficacy, and accessibility of therapy.

**Table 2:** A comparison of the risks, costs, efficacy, and accessibility of the lentiviral-based and CRISPR-Cas9-based gene therapies for the treatment of sickle cell disease.

	Lentiviral-Based Gene Therapy	CRISPR-Cas9-Based Gene Therapy
Gene Therapy Name	Lyfgenia ^TM^	Casgevy ^TM^
Regulatory approval	2023	2023
Genome editing tool	Zinc finger nucleases	CRISPR-Cas9 with single guide RNA
Viral-based	Yes, lentiviral	Nonviral
Risks of gene therapy		
- Due to myeloablative conditioning (chemotherapy)	Yes, present, including treatment-related mortality, infertility, and increased risk of cancer	Yes, present, including treatment-related mortality, infertility, and increased risk of cancer
- Secondary malignancy reported	Yes, acute myeloid leukemia was reported in two patients after gene therapy (see Section 2.3.2); FDA black box warning	No secondary malignancy has yet been reported
- Off-target toxicity risk present	Yes, due to possible viral vector insertion at any off-target site	Yes, due to genome edits at off-target sites
Costs of therapy	$3.1 million	$2.2 million
Efficacy of therapy	High chance of eliminating vaso-occlusive crises and disease symptoms	High chance of eliminating vaso-occlusive crises and disease symptoms
Accessibility	Only available for ages 12 years and older with recurrent vaso-occlusive events	Only available for ages 12 years and older with recurrent vaso-occlusive events
	Only available in specialized centers with access to expertise in hematopoietic stem cell transplantation	Only available in specialized centers with access to expertise in hematopoietic stem cell transplantation
Follow-up	Extensive follow-up required to evaluate long-term efficacy and safety	Extensive follow-up required to evaluate long-term efficacy and safety

FDA, Food and Drug Administration

#### The Potential Risks of Lentiviral-Based and CRISPR-Cas9-Based Gene Therapies

The risks of both gene therapies are related to myeloablative conditioning, with adverse effects of chemotherapy including treatment-related mortality, infertility, hair loss, mouth ulcers, increased risk of infection and bleeding, and increased risk of cancer. Fertility preservation options, such as freezing eggs or sperm, may be helpful [116]. The gene therapy does not change the DNA in the sperm or egg of the patient receiving gene therapy; therefore, the risk of transmitting the sickle cell variant allele to a child remains, and that risk determination requires knowing the partner’s carrier status, with genetic counseling [116]. Despite the risks of gene therapy, it has been shown that patients with severe sickle cell disease would prefer to undergo gene therapy for disease amelioration [117,118]. However, the risk of infertility can be a major deterrent for some patients and parents [119]. The increased risk of cancer after gene therapy is due to the concern for the possibility of viral integration in an off-target site for the lentiviral-based therapy. As discussed above, this was considered unlikely in the two patients who developed a secondary myeloid leukemia post-therapy.

The major concern of CRISPR-Cas9-sgRNA-based gene therapy is the potential for off-target activity, for which post-marketing studies and the recipient patients’ long-term safety were recommended with the FDA approval [115]. The manufacturers’ efforts to minimize and monitor off-target editing were explained in a publication in May 2024 [120] and merit some explanation here since these efforts are crucial to assessing the specificity of this gene therapy. The manufacturers noted the following points about this gene therapy [120]: (1) they had selected a unique on-target site that had low homology to the remainder of the genome, and (2) they had used “an ex vivo approach that allowed transient exposure to Cas9 and guide RNA only in hematopoietic lineage cells, as well as direct off-target evaluation” [120]. In this publication, the manufacturers summarized their studies, which did not identify any off-target editing in the examined coding sites in the genome that had been identified computationally to be studied, including in healthy donors and patients with sickle cell disease and transfusion-dependent thalassemia [120]. The investigators will continue monitoring the patients in the long term [120].

## 4. The Cost-Effectiveness of Gene Therapy Compared with Standard-of-Care Treatment for Sickle Cell Disease

Patients who receive gene therapy for sickle cell disease are expected to experience meaningful improvements in outcomes and quality of life. For those who received the lentiviral gene therapy, survival is predicted to increase by 23.84 years compared to common care in patients aged ≥12 years with ≥4 vaso-occlusive events in the past 24 months [121]. The life expectancy with gene therapy is expected to be 62.24 years versus 38.40 years for standard care [121]. In another study, a price tag below $2 million for gene therapy was considered likely to be cost-effective from a societal perspective [122].

## 5. Examples of Other Ex Vivo Gene Therapy Approaches for the Treatment of Severe Sickle Cell Disease

### 5.1. Ex Vivo Lentiviral-Based Approaches Studied or in Clinical Trials for Gene Therapy in Sickle Cell Disease

Another lentiviral approach induced high levels of fetal globin by reducing the mRNA for *BCL11A* by using short hairpin RNA embedded in a microRNA, enabling specific blockdown of the erythroid lineage [123]. All six patients enrolled in this trial engrafted and achieved stable HbF induction with reduced or absent symptoms of sickle cell disease [123].

Zinc finger nucleases have been developed to target and disrupt the erythroid enhancer of *BCL11A* in CD34+ HSPCs ex vivo to reactivate fetal hemoglobin [124]. BIVV003 is a ZFN-mediated autologous gene therapy in clinical development for the treatment of sickle cell disease, with the ZFN targeting a GATAA motif in the erythroid enhancer of *BCL11A* (NCT03653247) [125]. Interim results from these studies showed that BIVV003 produced long-term HSPCs capable of multilineage engraftment independent of mobilization strategy (plerixafor or plerixafor plus granulocyte colony-stimulating factor) or disease state and led to robust HbF induction in the red blood cells [125].

Another lentivirus-based phase 1/2 gene therapy trial used reduced-intensity conditioning for the autologous HSCT with genetically modified HSPCs to reduce toxicity and resource use. The trial enrolled seven patients with sickle cell disease, with reported 2–7 years of follow-up [126]. The viral vector in this study encoded a modified γ-globin gene expressing a potent anti-sickling fetal hemoglobin, HbF^G16D^. This variant was used since the investigators had hypothesized that the highly potent modified hemoglobin would help to achieve a clinical benefit that might otherwise be lower with reduced-intensity conditioning than with myeloablative conditioning [126]. The primary feasibility endpoints were met. Adverse events (total *n* = 503) occurred in seven patients throughout the study, with grade 2–3 vaso-occlusive crises being the most common, a 5-day median duration of grade 4 thrombocytopenia, and an 8-day median duration of grade 4 neutropenia. All seven patients showed sustained expression of HbF^G16D^, with >80% reduction in vaso-occlusive events [126].

### 5.2. Other Ex Vivo CRISPR-Cas9 Single Guide RNA-Based Approaches Studied for Gene Therapy in Sickle Cell Disease

This section discusses examples of other CRISPR-Cas9-based ex vivo genetic editing approaches that have been studied to treat sickle cell disease.

#### 5.2.1. Preclinical Studies to Mimic Hereditary Persistence of Fetal Hemoglobin (HPFH) or Introduce HPFH-like Mutations in CD34+ HSPCs

Hereditary persistence of fetal hemoglobin (HPFH) refers to a group of heritable disorders with increased levels of HbF that persist in adults. These disorders can occur due to deletions in the β-globin gene locus or nondeletional changes in the promoter region of γ-globin gene [127]. HPFH most commonly occurs due to deletions, and at least eight different types of deletional HPFH have been recognized [128,129,130]. Individuals with β-thalassemia or sickle cell disease with co-occurring deletional type of HPFH show a pancellular HbF distribution, with asymptomatic or mild disease. In a preclinical proof-of-concept study, CRISPR-Cas9-sgRNA-based genome editing was used to excise a 13 kb segment of the β-globin gene locus in bone marrow-derived CD34+ HSPCs to mimic a naturally occurring Sicilian type of HPFH (HPFH5) [130]. This approach led to genetic modification of 31% of the bone marrow CD34+ HSPCs, which showed significantly higher γ-globin expression in the erythroid cells derived from the modified HSPCs than the cells without the deletion [130].

In another preclinical study, CRISPR-Cas9-based genome editing was used to disrupt the promoters of *HBG1* and *HBG2* genes in patient-derived CD34+ HSPCs to mimic a naturally occurring HPFH-associated mutation. This approach induced HbF expression in the erythroid cells to potentially therapeutic levels [131].

CRISPR-derived base editing does not cause the double-stranded DNA break that occurs due to CRISPR-Cas9-based genome editing. Therefore, base editing has been used to introduce many point mutations into the *HBG* promoters, followed by screening for those mutations that induce HbF to therapeutic levels [132]. Adenine base editors (ABE) convert A-T to G-C, and cytosine base editors (CBE) convert C-G to T-A. Both types of base editors were highly efficient in creating point mutations in HSPCs; several point mutation targets were identified that could potentially be used for therapeutic purposes [132].

#### 5.2.2. Phase 1/2 Clinical Trial for Targeted Disruption of the HBG1 and HBG2 (γ-globin) Gene Promoters

In this clinical trial, three patients with sickle cell disease received CRISPR-Cas9-edited autologous CD34+ HSPCs after myeloablative conditioning [133]. The *HBG1* and *HBG2* (γ-globin) gene promoters in the transplanted HSPCs were disrupted by CRISPR-Cas9. The guide RNA for Cas9 had been previously identified by a tiling CRISPR-Cas screen of *HBG1* and *HBG2* promoters during preclinical studies [133]. The single gene therapy product infusion led to sustained increases in total hemoglobin and HbF and decreased vaso-occlusive crises, with no detected off-target effects [133]. Adverse events were related to myeloablative busulfan conditioning and sickle cell disease, not to the gene therapy product; alternative conditioning agents were suggested in future trials for reduced toxicity [133].

#### 5.2.3. Converting the Sickle Mutation to a Non-Sickling Globin Variant by Base Editing

In sickle cell disease, the normal GAG (Glu) codon encoding the 6th amino acid of β-globin is mutated to GTG (Val), forming *HBB*^S^. Since adenine base editors convert A-T to G-C base pairs in living cells, they cannot correct the pathogenic mutation in sickle cell disease to the normal allele. However, adenine base editors can convert the pathogenic codon GTG (Val) to GCG (Ala), which leads to a non-pathogenic hemoglobin termed Hb-Makassar (*HBB*^G^) [134].

A custom ABE [135] used to edit HSPCs derived from patients with sickle cell disease ex vivo converted 80% *HBB*^S^ to *HBB*^G^. Transplanting the edited HSPCs into immunodeficient mice showed durable editing, with a 5-fold decrease in hypoxia-induced sickling in bone marrow reticulocytes. Then, the custom ABE was used to edit HSPCs from a humanized sickle cell disease mouse. Those edited HSPCs were transplanted into irradiated mice to show a 3-fold reduction in hypoxia-induced sickling and the conversion of hematologic parameters to near-normal levels [134]. The edited bone marrow was secondarily transplanted to confirm durable editing of long-term HSCs [134].

The potential advantages of the base editing approach include: (1) preventing a double-stranded DNA break caused by CRISPR-Cas9 nucleases, (2) more effective reduction in HbS compared to approaches with lentiviral expression of β-globin variant or induction of HbF, since the latter approaches do not correct the HbS allele, and (3) no requirement for exogenous DNA delivery, which is required for homology-directed repair of the double-stranded DNA break by Cas9 nuclease [134].

#### 5.2.4. FDA-Approved Phase I/II Clinical Trials to Correct the Sickle Mutation in HSPCs by CRISPR-Cas9 Editing Using a Single-Stranded Oligonucleotide Donor by Electroporation or by an Adeno-Associated Virus (AAV) Vector

In November 2024, the FDA approved the first in-human clinical trial to correct the sickle mutation using CRISPR-Cas9 genome editing by a non-viral approach [136] (NCT04774536 https://clinicaltrials.gov/study/NCT04774536#study-overview, accessed on 15 September 2025). To correct the sickle cell mutation, new methods were required in this approach to produce more than 100 million gene-corrected, healthy cells per patient. This number is much higher than the 1–2 million gene-corrected cells produced by earlier methods. In this therapy, termed CRISPR_SCD001, the patient’s peripheral blood stem cells will be collected by apheresis and genetically modified by CRISPR-Cas9-sgRNA-based cleavage at the sickle mutation site and homology-directed repair using a single-stranded oligonucleotide donor [136,137]. The investigators avoided the use of an adeno-associated virus (AAV) donor template based on their own and other studies, which showed increased correction efficiency with an AAV donor, but also induced cytotoxicity that impaired engraftment [137,138,139,140]. Plerixafor was used for mobilization to avoid using granulocyte colony-stimulating factor (G-CSF) in patients with sickle cell disease. CD34+ HSCs that were homozygous for the sickle allele were collected by apheresis from an adult patient. Electroporation, which causes temporary pores in the cell membrane, was used to deliver the CRISPR gene editing platform components to reach the cell nucleus [137]. After the edited HSCs were transplanted in mice, the long-term HSCs showed >30% correction of the β-globin gene.

Notably, during erythroid differentiation in the xenografted mice, the erythroblasts with the corrected alleles were enriched, indicating that those erythroid cells had a survival advantage. This gene editing protocol created a population of cells carrying at least one normal *HBB* allele that would be sufficient for red blood cell function. The authors’ studies also found that *HBB* null alleles were deleted along with the enrichment of the corrected alleles. These findings were consistent with earlier observations after an allogeneic HSCT in sickle cell disease and β-thalassemia, wherein a donor contribution as low as 20% in the marrow results in dominant donor erythrocytes in the circulation [137]. The authors concluded that a gene editing protocol capable of producing >20% corrected HSCs (mono-allelic or biallelic correction) should be able to eliminate the symptoms of sickle cell disease. Since their studies showed >30% correction, the editing protocol was eligible for a clinical trial in patients with sickle cell disease [137].

The second FDA-approved clinical trial currently recruiting is CRISPR-Cas9-based but uses an adeno-associated virus instead of a non-viral approach (NCT04819841). It is based on the studies mentioned above [138,140].

#### 5.2.5. Reverting the Sickle Cell Allele to a Wild Type Allele by Prime Editing [141]

Prime editing is an advanced genome editing method developed following CRISPR-Cas9-based gene editing (reviewed in [142] and briefly explained in [56]). In 2023, an ex vivo prime editing strategy was shown to correct the sickle mutation in HSPCs from patients with sickle cell disease to a wild-type allele at frequencies of 15–41% in the HSPCs with minimal off-target editing [141]. The prime-edited HSPCs were transplanted into immunodeficient mice to show engraftment at 17 weeks with erythroid differentiation and sustained expression of *HBB^A^* in 42% of human HSPC-derived erythroblasts and reticulocytes. *HBB^A^*-derived adult hemoglobin was present at 28–43% of normal levels in the red blood cells, which now carried less sickle hemoglobin and resisted hypoxia-induced sickling. The advantages of this approach include not requiring double-strand DNA breaks, viral transduction, or donor DNA templates [141].

## 6. Why Would In Vivo Gene Therapy for Sickle Cell Disease Transform the Treatment of Patients with Sickle Cell Disease Worldwide?

The infrastructure and the expertise required for an HSCT are necessary for ex vivo gene therapies described above. However, neither is readily available in developing countries where sickle cell disease is most prevalent. Therefore, the clinical trial for Casgevy enrolled only patients from specialized centers in North America and Europe with access to experts in hematopoietic stem cell transplantation, as explained by the study investigators [143]. This significant obstacle could theoretically be overcome if the patients’ diseased hematopoietic stem cells could undergo genomic editing in vivo or directly within the patient without the need for the patient to undergo the process of an HSCT. Also, developing gene therapies where the disease is most prevalent could help patients in low-resource countries; developing affordable gene therapies was reported as the number three priority amongst India’s top three research priorities for sickle cell disease [144].

The following subsections briefly discuss significant preclinical studies and challenges to the goal of in vivo gene therapies. The most significant challenge for in vivo genome editing is delivering the genome editing components to the desired cells where the edits need to be made. In this respect, lipid-based nanoparticles (LNPs) are emerging as promising delivery vehicles, which are briefly explained.

### 6.1. Preclinical Studies Using an Adenoviral Vector System Toward In Vivo Hematopoietic Stem Cell Gene Therapy in Sickle Cell Disease

Toward the goal of in vivo HSC gene therapy to benefit patients worldwide, Li et al. first engineered a customized helper-dependent adenovirus (HDAd) vector system (HDAd5/35++) with a larger insert capacity than available with lentiviral and adenovirus vectors to be able to use a prime editing strategy to correct the sickle mutation [145,146]. They tested this system in mice [147]. Then, they showed in a proof-of-concept study in nonhuman primates that “in vivo gene therapy could be feasible in humans without the need for high-dose chemotherapy conditioning and HSC transplantation” [148].

In their 2023 study of in vivo HSC gene therapy in a sickle cell disease mouse model, they applied this vector system with a prime editing approach. The genome edits converted the mutant GTG (Val) codon to GAA (Glu) in approximately 40% of sickle alleles in the HSCs, with 43% of sickle hemoglobin replaced by adult hemoglobin [146]. Despite promising efficacy results, the authors discussed safety concerns due to the potential release of proinflammatory cytokines after intravenous injection of the HDAd viral vector, potential cytotoxicity due to the transduction of HSCs by HDAd, and the undesired editing of non-hematopoietic tissues by the HDAd vector system [146]. Subsequently, a G-CSF-free rapid stem cell mobilization protocol with in vivo prime editing showed efficacy in mice [149].

In a later publication [150], the authors reported further potential concerns regarding their 2023 prime editing in vivo study: (1) The target site efficiency of the prime editing approach was low at 5% when tested in vitro in CD34+ cells obtained from sickle cell disease patients. (2) The HDAd viral vector system with the prime editing components transiently expressed a dominant-negative *MLH1* gene, potentially increasing genotoxicity if *MLH1* were suppressed, since *MLH1* is involved in DNA repair. (3) The prime editing components include retroviral reverse transcriptase, a potential safety concern [150].

Then, the authors used the custom adenine base editor and the sgRNA used in an earlier study to correct the sickle mutation to the Makassar variant [134] (described in Section 5.2.3 above). They combined the custom editor with the HDAd vector. They showed that this HDAd-base editor vector system corrected the sickle cell mutation (GTG) to the benign Makassar variant (GCG) in vitro in CD34+ HSCs from sickle cell disease patients and in vivo in mice [150]. This in vivo study led to a 24.5% Makassar variant in long-term HSCs in the sickle cell disease mice. The in vivo HSC transduction approach in this study [150] involved a one-time intravenous infusion of the HDAd-adenine base editing vector system into mice that had their HSCs mobilized by a subcutaneous injection of G-CSF, followed by AMD3100 [150,151]. To expand the genetically edited HSCs, low doses of O6-benzylguanine (O6-BG) and 1,3-bis(2-chloroethyl)-1-nitroso-urea (BCNU) [152] (O^6^BG/BCNU) were given [150]. The mice were observed for 16 weeks, followed by isolation of Lin-negative bone marrow cells. Without the O^6^BG/BCNU selection, the sickle mutation conversion rate was only 3.0% (range 2.1–4.8%) in peripheral blood mononuclear cells (PBMCs), and 3.5% in Lin-negative bone marrow cells at week 10 after transduction.

In contrast, with O^6^BG/BCNU selection, the PBMCs showed a conversion rate of 23.8% (range 18–33%) of the sickle mutation at week 6 and 25.4% at week 16. There were comparable editing rates in the spleen, bone marrow mononuclear cells, and Lin-negative cells, pooled colonies from Lin-negative cells, and cells arising from non-erythroid lineages, including T and B lymphoid cells, and myeloid cells, indicating that gene modification had occurred in multipotent HSPCs [150]. Editing in non-hematopoietic tissues was also observed at 16 weeks post-transduction, with rates of 19.6% in the liver, 22.4% in the lung, around 10% in the heart, kidney, and intestine, and 3.4% in the brain, with no editing detected in the ovaries in the female mice [150].

### 6.2. Lipid-Based Nanoparticles for In Vivo CRISPR-Based Gene Editing, Including for Hematopoietic Stem Cells

Lipid-based nanoparticles have been researched as promising non-viral drug delivery vehicles since the first FDA approval given in 2018 to LNPs to deliver a small interfering RNA (siRNA), which was used in the clinical trial for in vivo CRISPR-based gene editing for transthyretin amyloidosis [153,154]. The FDA also approved LNPs as the vehicle to deliver mRNA vaccines to protect from coronavirus disease-2019 [155]. As discussed in a recent review [156], LNPs have several advantages over using AAV vectors as drug delivery vehicles for in vivo genome editing. These include less immunogenicity and a lesser risk of insertional mutagenesis than AAV vectors, and the potential for redosing is available with LNP-based delivery vehicles [156].

For genome editing of HSCs in vivo, a promising study in 2023 showed the successful use of an in vivo base editing system in mice. That system was delivered in vivo by a single injection of LNPs containing mRNA targeted against the stem cell receptor CD117, which is present on the surface of HSCs [157]. In addition, pro-apoptotic PUMA (p53 up-regulated modulator of apoptosis) mRNA delivered in vivo through the LNP could avoid the pre-HSCT toxic conditioning required to kill the pre-existing bone marrow cells [157].

An additional study was presented at the 2024 American Society of Hematology meeting for in vivo correction of the sickle mutation by using RNA gene writers [158]. This non-viral and non-CRISPR-based gene editing technology uses target-primed reverse transcription and RNA-containing LNPs delivered in vivo. The study showed the desired installation of the Makassar β-globin variant in more than 30% of long-term-HSCs with a single dose of their proprietary LNPs given to immunodeficient mice engrafted with healthy donor CD34+ cells. A second dose of the LNPs showed a linear increase in the percentages of edited long-term HSCs, with >70% gene rewriting observed in HSPCs and long-term HSCs in mice; similar results were shown in non-human primates [158].

Notably, LNPs were used as the delivery vehicle in the recently reported successful clinical application of base editing in an infant born with a rare genetic disease [159]. Long-term follow-up is necessary for evaluating the safety and efficacy of this treatment. Still, this unprecedented advance, accomplished within seven months after the infant’s birth and which could be applied to many other diseases, is a testament to the rapid pace of research in this field, providing hope for future breakthroughs to benefit patients.

## 7. Conclusions

The incidence and prevalence of sickle cell disease are rising globally, with the highest disease burden in low- and middle-income countries. The disease also has a high global mortality burden. Intensive global efforts are ongoing to improve the outcomes for patients with sickle cell disease significantly, emphasizing simple measures such as early diagnosis, ideally by newborn screening, treatment with hydroxyurea and blood transfusions, vaccinations, and infection prophylaxis, which can reduce morbidity and mortality. Allogeneic hematopoietic HSCT was the only curative treatment available until 2023, when two one-time curative ex vivo gene therapies, Lyfgenia and Casgevy, were approved for treating patients with sickle cell disease. Tremendous research for more than three decades towards successful human gene therapy led to these landmark gene therapy approvals to treat sickle cell disease. Nevertheless, both allogeneic HSCT and ex vivo gene therapies require an infrastructure and special expertise for an HSCT, which are not readily available in most low- and middle-income countries, where the disease is most prevalent. Thus, these curative therapies are available only to developed and resource-rich countries with HSCT expertise. Research is ongoing to prevent the potential adverse effects of the pre-HSCT conditioning required for both HSCT and ex vivo gene therapies. Long-term follow-up for at least 15 years is required to evaluate the efficacy and safety of gene therapies. Developing more ex vivo gene therapies in the countries where the disease is prevalent would benefit patients in low-resource settings.

Preclinical research is also being conducted on in vivo gene therapy, which could prevent patients from undergoing the HSCT procedure and increase curative treatment access for numerous patients. These early-phase approaches still face challenges and hurdles to be overcome. However, the pace of research advances has been rapid and unprecedented in the last decade. Even if in vivo genome editing platforms become fully developed for clinical curative treatment a decade or two from now, in vivo gene therapy would have the potential to transform the treatment landscape and make it possible to cure patients with sickle cell disease even in high-burden low-resource settings and low- and middle-income countries.

## Data Availability

No new data were created or analyzed in this study. Data sharing is not applicable to this article.

## Abbreviations

The following abbreviations are used in this manuscript:

AAV	Adeno-associated virus
ABE	Adenine base editor
BCNU	1,3-bis(2-chloroethyl)-1-nitroso-urea
Cas	CRISPR-associated
CI	Confidence interval
CIBMTR	Center for International Blood and Marrow Transplant Research
CRISPR	Clustered, regularly interspaced, short, palindromic repeats
EBMT	European Blood and Marrow Transplant
FDA	Food and Drug Administration
G-CSF	Granulocyte colony-stimulating factor
GVHD	Graft versus host disease
HbS	Hemoglobin S
HDAd	Helper-dependent adenovirus
HLA	Human leukocyte antigen
HSCs	Hematopoietic stem cells
HSCT	Hematopoietic stem cell transplant
HSPCs	Hematopoietic stem and progenitor cells
HPFH	Hereditary persistence of fetal hemoglobin
LNPs	Lipid-based nanoparticles
NBS	Newborn screening
O6-BG	O6-benzylguanine
PBMCs	Peripheral blood mononuclear cells
sgRNA	Single-guide RNA
TALE	Transcription activator-like effector
TALEN	Transcription activator-like effector (TALE) nuclease
ZFNs	Zinc finger nucleases
